# Enhanced Rabies Surveillance to Support Effective Oral Rabies Vaccination of Raccoons in the Eastern United States

**DOI:** 10.3390/tropicalmed2030034

**Published:** 2017-07-28

**Authors:** Jordona D. Kirby, Richard B. Chipman, Kathleen M. Nelson, Charles E. Rupprecht, Jesse D. Blanton, Timothy P. Algeo, Dennis Slate

**Affiliations:** 1 United States Department of Agriculture, Animal and Plant Health Inspection Service, Wildlife Services, National Rabies Management Program, Concord, NH 03301, USA; richard.b.chipman@aphis.usda.gov (R.B.C.); kathleen.m.nelson@aphis.usda.gov (K.M.N.); timothy.p.algeo@aphis.usda.gov (T.P.A.); dennis.slate@aphis.usda.gov (D.S.); 2 LYSSA, LLC, Atlanta, GA 30333, USA; charleserupprechtii@gmail.com; 3 Poxvirus and Rabies Branch, Division of High-Consequence Pathogens and Pathology, National Center for Emerging and Zoonotic Infectious Diseases, Centers for Disease Control and Prevention, Atlanta, GA 30333, USA; asi6@cdc.gov

**Keywords:** enhanced rabies surveillance, oral rabies vaccination, rabies elimination, raccoon, wildlife, zoonosis

## Abstract

Enhanced rabies surveillance (ERS) is essential for sound oral rabies vaccination (ORV) decisions to prevent the spread of specific rabies virus variants in meso-carnivores and to achieve disease elimination. Use of a direct rapid immunohistochemistry test (dRIT) in North America for timely, accurate rabies diagnosis in the field has facilitated greater ERS emphasis since 2005. ERS used in tandem with exposure-based public health surveillance provides a comprehensive understanding of the geographic distribution of rabies as an aid to formulate effective management strategies for raccoons and other meso-carnivores. In 2015, best management practices were implemented for improving, reinvigorating, and standardizing ERS. A point system for weighing ERS sample categories was evaluated, to determine whether sampling emphasis should be focused upon ill or strange-acting animals, the highest quality category. During 2016, 70.7% of rabid animals detected through ERS in raccoon rabies management states were obtained from strange-acting animals, followed by animals found dead (14.1%), road kills (9.1%), and nuisance-collected specimens (6.1%). Sample category weights may be adjusted based on additional evaluation to ensure continued emphasis on the highest value samples. High quality ERS, in conjunction with serologic evidence of population-based immunity, form the backbone for ORV decisions in the elimination of raccoon rabies.

## 1. Introduction

Surveillance is the cornerstone in providing actionable data for effective wildlife disease management. Knowing specifically when and where disease occurs is vital to formulate prevention, control and elimination strategies. The World Organisation for Animal Health (OIE) has endorsed prevention of rabies in source populations [1], which includes terrestrial meso-carnivore species in North America such as raccoons (*Procyon lotor*) and striped skunks (*Mephitis mephitis*). Oral rabies vaccination (ORV) has enabled a shift in rabies management focus to the source of disease in wildlife reservoirs. The use of ORV has proven successful in eliminating rabies in European foxes [2], and multiple terrestrial reservoirs in Canada [3,4,5]. In the U.S., considerable emphasis has been focused on preventing the spread of the raccoon rabies virus variant in the eastern part of the country by creating vaccination barriers ahead of the disease front [6].

To achieve the goal of controlling and eliminating rabies in terrestrial wildlife, ORV is indispensable. Yet, without near-real time surveillance to delineate where ORV should be applied, its effectiveness at a landscape scale is doubtful. Diverse meso-carnivore hosts for many of the documented rabies virus variants in the U.S. further underscore the value of prompt rabies virus detection, variant typing and timely, accurate mapping of cases by virus variant and species.

In the U.S., rabies surveillance continues to be largely exposure-based in relation to public health [7]. Public health surveillance, often used interchangeably with passive surveillance, implies rabies detection without an active effort targeting a specific area or species to search for the disease [8]. While this system has been, and continues to be, effective in protecting human and animal health, exposure-based surveillance often may not adequately characterize the spatial-temporal distribution of rabies in wild meso-carnivores in real-time for effective intervention. Public health surveillance tends to be biased by human population density, and often discounts potentially rabid animals if no human or domestic animal exposure has occurred [6]. 

In recognition of this limitation in relation to ORV, a paradigm shift took place in the U.S. during 2004, where enhanced rabies surveillance (ERS) was initiated to complement public health surveillanceto provide a more comprehensive picture of rabies distribution by virus variant [6,9]. ERS is planned targeted surveillance that emphasizes a special effort to meet specific management needs. ERS is frequently used interchangeably with active rabies surveillance, is targeted, preferably based on risk factors, and is typically initiated with a designed sampling scheme to meet specific disease management needs [8]. This shift was facilitated largely through the application of a field-efficient, direct immunohistochemical test (dRIT) [10,11] by trained wildlife biologists in collaboration with the Centers for Disease Control and Prevention (CDC, Atlanta, Georgia, GA, USA); the World Health Organization (WHO) Collaborating Centre, Wistar Institute (Philadelphia, Pennsylvania, PA, USA); and state health departments. 

The United States Department of Agriculture (USDA), Animal and Plant Health Inspection Service (APHIS), Wildlife Services (WS), National Rabies Management Program (NRMP; hereafter WS) conducts ERS in support of national rabies management goals that are focused on preventing the spread of, and ultimately eliminating specific rabies virus variants in meso-carnivores. The range of ERS samples collected includesthe following categories: suspect, ill or strange-acting animals without a rabies virus exposure record (hereafter, strange-acting), animals found dead and not associated with highway mortality, road kills; animals captured from focal areas where rabies cases have occurred recently (hereafter, surveillance-trapped), and apparently healthy animals collected through nuisance wildlife control operators (NWCO) or submitted by homeowners [6].

The value of ERS to raccoon rabies management was illustrated in highly urban-suburban northeastern Ohio during 2007. Public health surveillance rabies cases had been declining from 2004–2006 in an area where emergency ORV and trap-vaccinate-release rabies management strategies were ongoing, as part of a contingency response to an epizootic that began in 2004 [6,9]. All rabid animals were detected through ERS during 2007, highlighting its importance as a complement to public health surveillance to make informed rabies control decisions [9]. 

With a programmatic transition towardsthe elimination of raccoon rabies in the eastern U.S., the absence of cases will serve as the key metric to measure success in tandem with rabies virus neutralizing antibody (rVNA) sero-prevalence as an index to vaccine-induced herd immunity.These metrics form the basis to make decisions to move ORV zones farther into raccoon rabies enzootic areas. During 2015, a new U.S. program initiative was developed to standardize best management practices (BMPs) and reinvigorate ERS. The program was implemented in four pilot states to expand the diversity of surveillance collaborators and to enhance detection of strange-acting animals, the highest valued ERS sample category [9]. The program was expanded during 2016 to 16 rabies management states that implemented some components of the new ERS initiative with full ERS implementation by these statesin 2017. This revitalized ERS system includes well-defined categories to track sources of samples combined with a stratified point value system to weigh the quality of samples collected and tested. The point system and a quarterly point threshold were established to provide incentive to collect the highest quality ERS samples for detection of rabid animals. This system also provides a platform for evaluating and adjusting ERS performance in participating states, and serves as a means for making adjustments in surveillance sampling emphasis over time.

The objective of this paper is to summarize the first year of categorized ERS data from 16 eastern states seasonally and annually. In addition, we categorized ERS retrospective data from the four pilot states during 2015, for comparison to 2016, within the context of the value of ERS data for planning and implementing raccoon rabies elimination. 

## 2. Materials and Methods 

During 2015, WS developed BMPs for ERS to integrate four primary components into an improved, adaptive approach to surveillance: (1) an established diverse, cooperative surveillance networking matrix; (2) an algorithm for strange-acting animals, sample prioritization, and freezer support for temporary sample storage; (3) rabies laboratory support; and (4) data entry, monitoring and analyses.

Alabama, Maine, Ohio and West Virginia were selected as pilot states to lead implementation of the ERS initiative in 2015, with a focus on establishing a diverse, cooperative surveillance networking matrix. Potential network links were represented by federal, state, county, and municipal agencies, special interest groups, the private sector, national organizations having regional and state affiliates, and international collaborators (Table 1). A recommended minimum number of contacts for each cooperator level was established to ensure diversity of cooperators within the network. Pilot states were selected, in part, on the range of their strategic rabies management history and current value to national ORV goals. In addition, these states were selected because their existing ERS strategies were not standardized prior to 2015. The purpose of establishing and maintaining a diverse, cooperator-based network was to increase the likelihood of collecting high quality samples from suspect animals that displayed behaviors suggestive of rabies.

**Figure 1:**
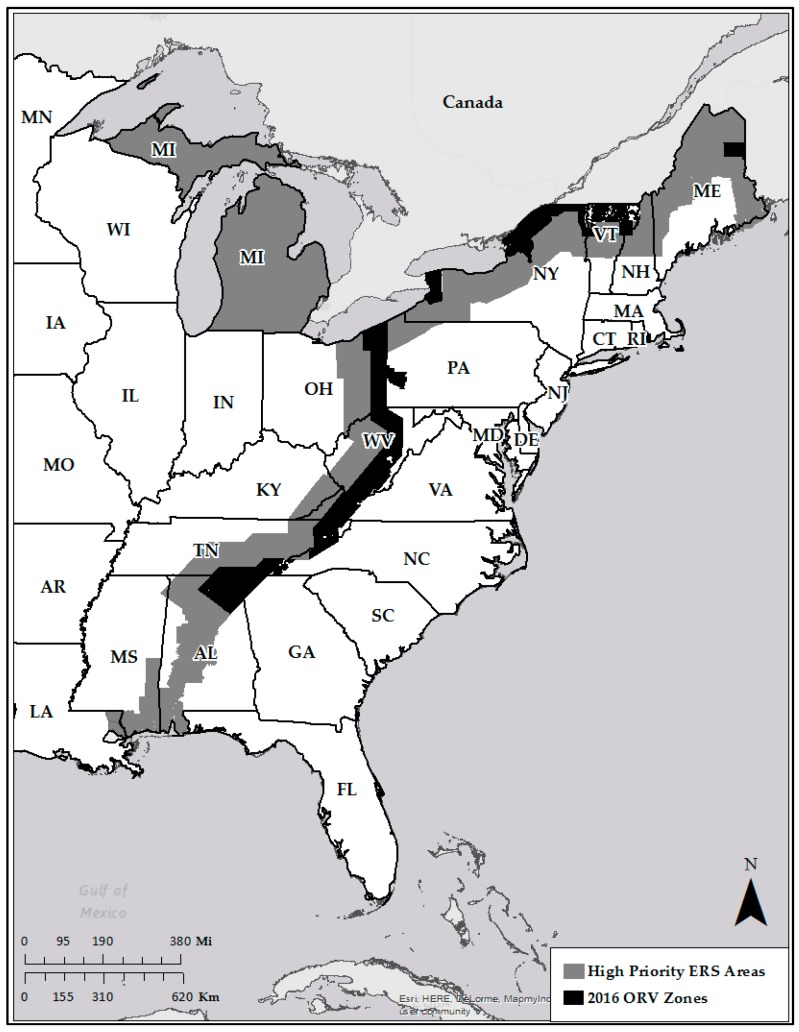
Highest priority enhanced rabies surveillance areas that extended 80 km west (Appalachian Ridge) or generally south (northeastern states) of current oral rabies vaccination (ORV) zones in the eastern U.S. and other critical risk areas.

Historically, ERS emphasized areas within and on the leading edge of ORV zones, where the risk of rabies spread beyond existing barriers was assumed to be high [12]. During 2015, the ERS area was formally designated to include the western half (20 km) of ORV zones, and the area extending 80 km west from the edge of current ORV zones along the Appalachian Ridge mountains, a 100 km-wide priority ERS focus area (Figure 1). In states bordering Canada (Maine, ME, New Hampshire, NH, New York, NY, and Vermont, VT, USA), the highest priority areas were identified as the existing ORV zones, which represented a ≥40 km wide vaccination zone along the USA-Canadian border, and 80 km south of the existing ORV zones. Other critical areas were also defined, including peninsular Cape Cod, Massachusetts, and potential risk corridors with a perceived increased likelihood of rabies spread in Alabama, Kentucky, Louisiana, Michigan, and Mississippi.

We defined 6 standardized ERS sample categories within a stratified point system to value samples according to a preliminary expectation of meso-carnivore specimens that might test positive for rabies antigen (Table 2) [9]. Initial point values were devised, based on an interpretative review of ERS category data from Ohio during 2005–2007, which illustrated that strange-acting animals had a higher likelihood of being rabid compared to all other ERS categories by 6–35 times [9]. Thus the point value assigned to this category was three-fold greater than the next most valuable category, and 15 times greater than the least valuable categories. In lieu of mandating sample sizes for ERS, we initially established a minimum threshold value of 100 points/quarter beginning in January 2016. This initial baseline threshold was designed to provide incentives for the collection of high quality surveillance samples evenly through time, as well as a guide to ensure a minimum consistent effort. It was not designed to account for seasonal spikes in rabies incidence; rather, it was designed to refocus ERS efforts on more consistent sample collection throughout the entire year.

We evaluated the stratified point system by assigning one of the six defined sample categories to all ERS samples collected by WS in 16 raccoon rabies management states, 1 January 2016–19 March 2017 (Figure 1). We evaluated data by calendar year (1 January–31 December 2016) and by calendar season (20 March 2016–19 March 2017). Calendar seasons were defined as follows: spring 2016 = 20 March–19 June 2016, summer 2016 = 20 June–21 September 2016, fall 2016 = 22 September–20 December 2016, and winter 2017 = 21 December 2016–19 March 2017. For each sample acquired through ERS, the minimum data collected included: date of sample collection, species, location (county-level or finer spatial scale), and behavior (if observed).

We used a univariate regression analysis to evaluate the relationship between rabid animal detection and each of the six sample categories for 16 states in calendar year 2016. To compare the categorized data in the four pilot states (Alabama, AL; Maine, ME; Ohio, OH; West Virginia, WV; USA) that implemented this surveillance system during calendar year 2015, we retrospectively assigned sample categories to all ERS samples collected in the four pilot states from 1 January–31 December 2015.

An algorithm was established to define the procedure for response to telephone calls received by cooperators regarding strange-acting animals. The algorithm provides a call-tree system to triage incoming calls from the general public about potentially rabid wildlife. Call algorithms were adapted in each state, dependent on cooperative entities within county or municipal areas responsible for responding to calls from the public. For example, a county health department that fielded a call about a suspect animal with no exposure might refer the caller to their respective game warden, whom in turn would dispatch the animal and submit it to WS. 

A network of freezers was established in states or expanded in several states (e.g., Ohio, OH and Tennessee, TN, USA) that had previously distributed freezers in strategic locations for sample submission and temporary sample storage prior to rabies testing. Freezer locations included county health departments, municipal animal control offices, and department of transportation facilities. The BMPs recommendation was for freezer samples to be collected a minimum of twice monthly and subsequently tested within a week when practical, with an expectation that high priority samples would be tested within 48 hours of collection.

Laboratory support outlined through the program initiative included state-level reaffirmation of cooperative relationships with state rabies diagnostic facilities. At the national programmatic level, WS engaged with the CDC, The Wistar Institute and the New York State Department of Health (NYSDOH) to ensure continued diagnostic support for confirmation of WS-tested dRIT samples using the gold standard direct fluorescent antibody (dFA) test, variant-typing for all rabid animal specimens, and availability of non-commercial monoclonal antibodies for the dRIT [6]. All dRIT positive and indeterminate specimens, in addition to 10% of negatives, were confirmed using the dFAtest. Once confirmed and variant-typed, all rabid specimen reports were provided to the appropriate cooperators in the surveillance network, though only raccoon rabies virus variant cases had implications for ORV.

The BMPs also outlined improved mechanisms for data entry, monitoring and analyses, including structured timelines for entering data into the WS Management Information System, quality assurance practices for data management, and guidelines for logging information to track cooperator network contact events to maintain a viable network over time. Improvements to data management also included assigning and recording ERS sample categories for every specimen collected and tested, which were not recorded formally prior to 2016.

## 3. Results

From 2005–2016, approximately 99,991 ERS samples were collected and tested through WS surveillance in 26 states and Puerto Rico, including 19 eastern states and seven states west of the Mississippi River. Rabid animals detected through ERS comprised 2.1% of all samples (*n* = 2107). Approximately 82.0% of all ERS samples were tested by WS using the dRIT and 72.1% of all ERS positives were detected using this field diagnostic method.

From 1 January–31 December 2016, ERS resulted in collection and testing of 6852 ERS samples from 16 raccoon rabies management states (Table 3), and 99 rabid animals were identified. These ERS samples generated a total of 27,851 ERS points (Table 3), with rabid animals representing 1153 ERS points. The sample assigned the highest point value, strange-acting wildlife, accounted for 18.1% of all samples collected and represented 66.7% of all weighted samples (Table 3). Strange-acting animals accounted for 70.7% of rabid animals detected through ERS, followed by 14.1% found dead, 9.1% road kills, and 6.1% nuisance-collected specimens. No rabid animals were detected in the surveillance-trapped or unknown categories. Rabies-positive samples from strange-acting animals represented 5.7% of all samples collected within this respective category, followed by 6.2% for animals found dead, 0.7% for road kill, none for surveillance-trapped, 0.2% for NWCO/other, and none for the unknown category.

Simple linear regression revealed a highly significant relationship (*p* < 0.0000002) between road kill samples collected and rabid meso-carnivores detected in 2016 (r^2^ = 0.86). A significant relationship (*p* <0.03) also occurred between animals found dead (not as road kills) and rabid meso-carnivores when data for Michigan (42 meso-carnivores found dead, with none testing rabies positive) were removed as a potential outlier, although the found dead sample category was not highly predictive (r^2^ = 0.32). The strange-acting sample category was weakly related to rabid meso-carnivores with New York data removed (63 ill or strange acting, with 19 testing rabid) as a potential outlier (0.07 < *p* < 0.05; r^2^ = 0.23). We suspect that a high ratio of rabid meso-carnivores were detected in New York in this category because WS may more carefully screen suspect animals submitted to the NYSDOH based on their recommendations for dFA testing rather than conducting dRIT.

**Figure 2:**
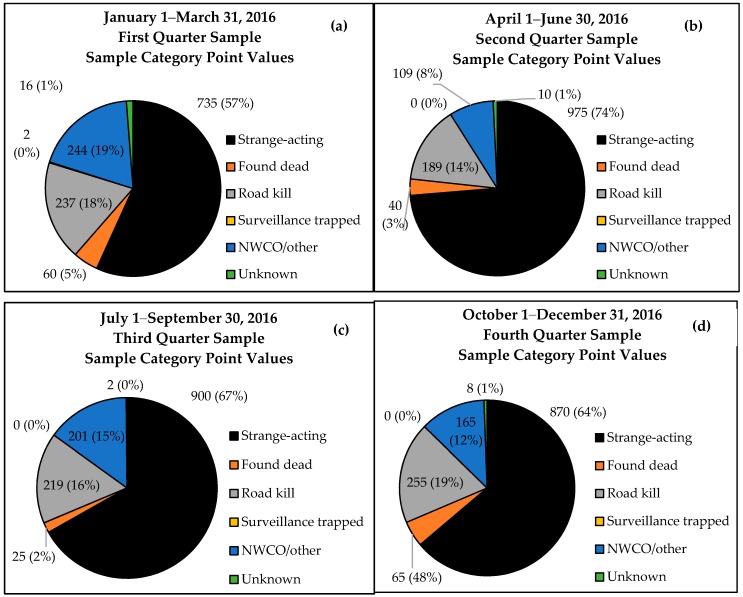
Minimum threshold weighted points and percentages by sample category for 16 states during (**a**) Quarter 1; (**b**) Quarter 2; (**c**) Quarter 3; and (**d**) Quarter 4 in 2016.

Twelve of 16 states achieved the ≥100 point minimum quarterly threshold except during the first quarter of 2016, where 10 of 16 states met or exceeded the minimum sample size target. Strange-acting samples accounted for 56.8–73.7% of threshold points by quarter, followed by road kill (14.3–18.7%), NWCO (8.2–18.9%), and animals found dead other than road kill (1.9–4.8%) (Figure 2).

**Figure 3:**
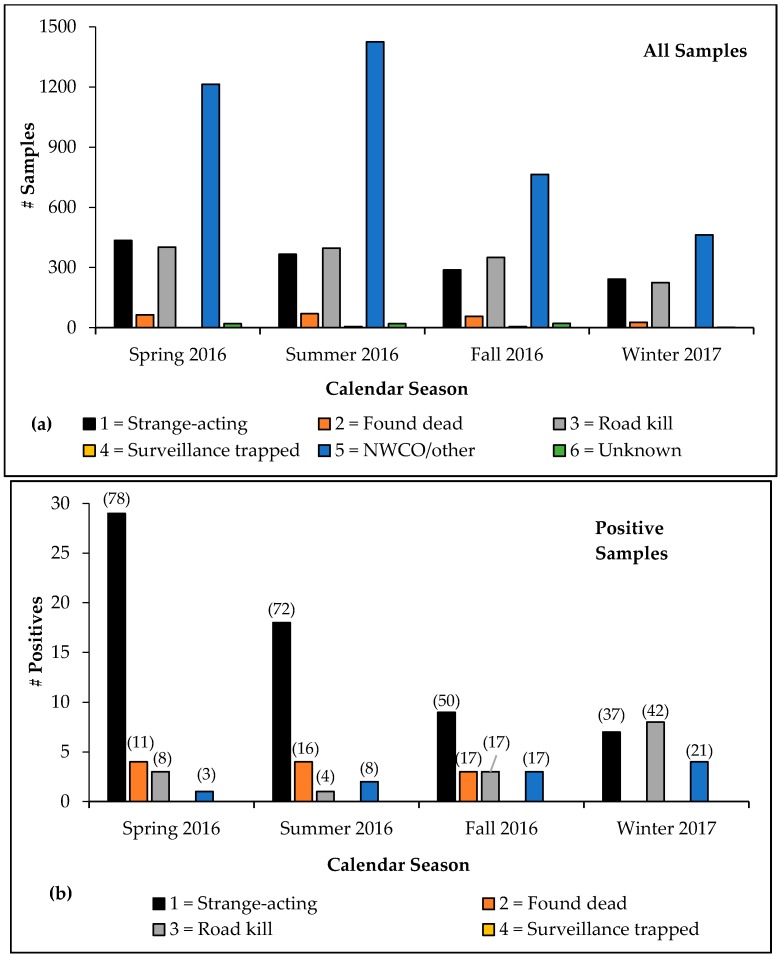
Comparison of enhanced rabies surveillance samples collected by season and sample category by Wildlife Services, spring 2016–winter 2017: (**a**) All samples and (**b**) Rabies-positive samples (value represents percent of all rabid animal samples detected according to sample category each season).

The highest volumeof samples were collected during summer 2016 (*n* = 2284), followed by spring 2016 (*n* = 2135), fall 2016 (*n* = 1486), and winter 2017 (*n* = 936). Samples from strange-acting animals were collected most frequently during spring 2016, followed by summer 2016, fall 2016, and winter 2017 (Figure 3a). The greatest proportion of rabid animal samples across all categories was detected during spring 2016 (*n* = 37 of 2135 samples), followed by summer 2016 (*n* = 25 of 2284), winter 2017 (*n* = 19 of 1486), and fall 2016 (*n* = 18 of 936; Figure 3b). The strange-acting category comprised the greatest proportion of rabid animals detected in all seasons except for winter 2017, during which road-killed animals represented the greatest proportion of rabies positives (Figure 3b). During fall 2016, animals found dead, road kills, and NWCO/other collected samples each represented 17% of rabid animal specimens. The NWCO/other positive samples represented a greater proportion of rabid animal samples during winter 2017 than those found dead, surveillance-trapped, and unknown categories. No rabid animals were detected from the surveillance-trapped or unknown categories during any season.

**Figure 4:**
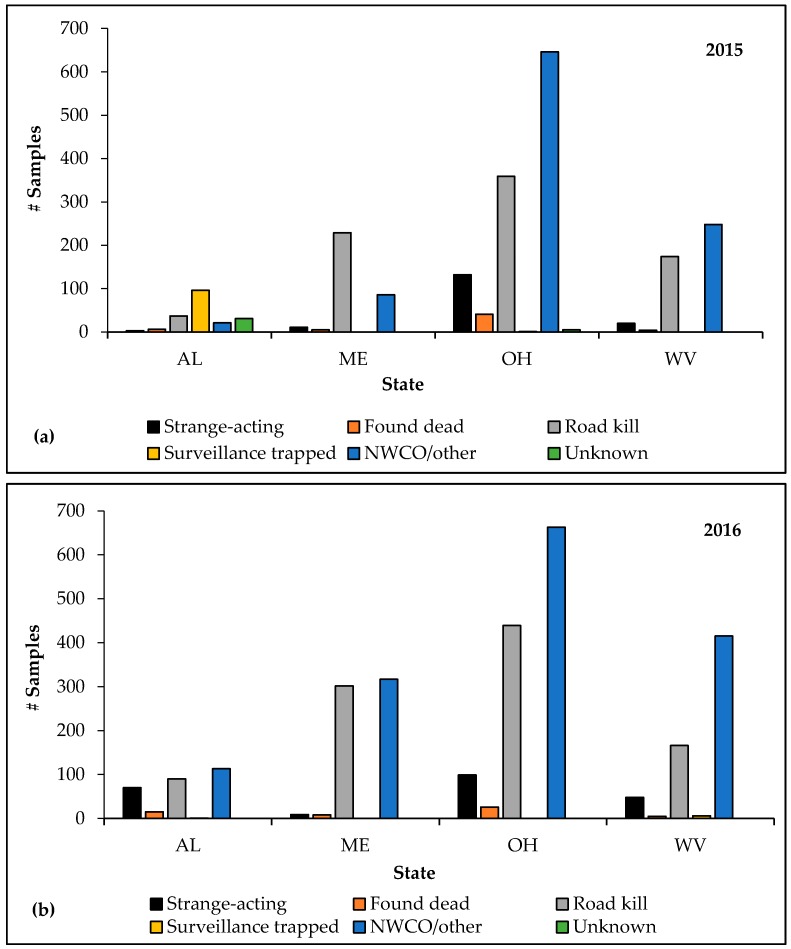
Comparison of enhanced rabies surveillance samples collected in 4 pilot states by Wildlife Services by sample category: (**a**) 1 January 2015–31 December 2015 and (**b**) 1 January 2016–31 December 2016. AL = Alabama; ME = Maine; OH = Ohio; WV = West Virginia.

Alabama, Maine, Ohio and West Virginia, which implemented the new ERS initiative during 2015, increased ERS sample collection by 29.6% (and category points by 27.7%) following full implementation in 2016. Specimens from strange-acting animals increased in two of four of these pilot states in 2016 compared to 2015, representing a 36.1% increase in sample collection within this category (Figure 4a,b). Ohio collected 33 fewer samples from strange-acting animals during 2016 than 2015, and Maine collected two fewer samples from strange-acting animals during 2016 than 2015. During 2015, rabid animals (*n* = 13) were reported in all pilot states except Alabama; all four pilot states reported rabid animals (*n* = 19) during 2016. Strange-acting animals represented 38.5% and 47.4% of positive specimens during 2015 and 2016, respectively, in the pilot states.

Maine had the greatest increase in ERS samples among the fourpilot states in 2016 compared to 2015, at 92.1%. Alabama, West Virginia, and Ohio increased total sample collection by 49.0%, 43.5%, and 3.1%, respectively. Alabama had the largest increase in ERS in total points and collection of the highest quality specimens among the four pilot states in 2016 compared to 2015. In Alabama, category points increased by 251%, from 430 in 2015 to 1510 in 2016 and collection of strange-acting specimens increased by 2233%, from three (2015) to 70 in 2016. Categorical points increased in Maine and West Virginia by 59.4% and 53.2%, respectively, but decreased in Ohio by 8.3%. Maine and West Virginia increased collection of the highest quality specimens by 72.7% and 140.0%, respectively, while Ohio decreased collection of specimens from strange-acting animals by 25.0%.

## 4. Discussion

An ERS program has been in place in the U.S. since 2004, and has been recognized as an effective means to increase sampling intensity and geographic scope for rabies virus detection when applied in tandem with public health surveillance [9]. A routine annual finding of approximately 2% (*n* = 176/year) of rabid animals detected directly through ERS from 2005 to 2016 near ORV zones is evidence of the value of ERS as a complement to public health surveillance. These rabid animals would not likely have been detected through exposure-based surveillance, and help to provide a more complete spatial and temporal picture of rabies virus distribution at a landscape level for improved management decision-making. A basic tenet of this surveillance paradigm shift beginning during 2004 was to promote more effective management of rabies at its source in reservoir species by focal detection, rather than mere increased testing of inappropriate or low-value specimens. Through the strategic application of ORV, there has been demonstrated success in preventing appreciable spread of the raccoon rabies virus variant in the eastern U.S. Clearly, ERS serves as a foundational component for managing rabies in wildlife reservoirs given that the absence of cases represents the ultimate measure of ORV success. 

Experimental access to an additional oral rabies vaccine, capable of producing higher indices of population immunity based on rVNA sero-prevalence post-ORV, has prompted WS to shift from containment to elimination strategies, underscoring the need to develop a more effective, formalized categorical sampling regime for ERS. Limitations associated with historic ERS implementation strategies were identified in 2015, including non-standardized tactics among state programs. Not all states participating in raccoon rabies management were focused on the collection of high quality samples, and instead collected primarily nuisance or otherwise apparently healthy animals. Furthermore, sampling did not occur uniformly in all ERS emphasis areas within and on the leading edge of ORV zones, potentially resulting in critical spatial and temporal ‘surveillance holes’ through which rabies could spread without timely detection. One such temporal surveillance gap observed was associated with a limited number of samples collected during winter months, despite programmatic evidence to suggest that meso-carnivores that are strange-acting, found dead, or killed along highways during winter in northern latitudes may have a higher likelihood of being rabid. Implementation of standardized ERS with BMPs that emphasize establishment and maintenance of a diverse cooperator network and that employs a stratified point system for weighing the quality of sample sources, served as the foundation for revitalizing the ERS system. 

Prior to 2015, WS coordinated primarily with the state departments of health, agriculture and wildlife on ERS. Through this ERS initiative, a diversity of other sources of suspect samples have been added to the historic suite of state agency collaborators, through strategic in-person meetings, telephone calls, mass mailings, and email blasts. A recommended minimum number of each contact type (e.g., federal, state, city/town, special interest, etc.) was provided as part of the BMPs (Table 1). Sample categories have been considered in ERS by WS since 2004, however, they were not formally defined for evaluation or put into a point system framework until the implementation of this ERS initiative during 2016. A weighted surveillance approach employing a stratified point system has been developed for other wildlife diseases, such as chronic wasting disease [13]. Point systems may improve ERS efficiency by stratifying sample collection according to biologically-relevant categories that may increase the probability of detecting a rabid animal [14]. A primary emphasis for the ERS point system was to create incentive by giving higher weight to higher quality ERS samples that have a greater chance for rabies virus detection. Initial point values were established based on an evaluation of ERS category data from Ohio [9] as well as historical WS ERS data collected over time. 

An initial quarterly point minimum threshold of 100 was established, based on historic ERS data trends. The threshold was designed to provide additional motivation to reach the 100 point mark more easily by focusing on the collection of high quality samples, but states were advised that this was only a minimum threshold and once reached, sampling should continue at the same pace. The one-year categorized snapshot of data suggests that the 100 point minimum was exceeded in most participating state programs through emphasis on higher quality ERS samples. A comparison of the point values for the four pilot states from 2015 to 2016 further highlights that the minimum threshold provided incentive to focus sample collection efforts on high quality specimens. Points within this sample category increased in all pilot states except for Ohio, and collection of strange-acting animals improved overall. These initial values serve as a baseline from which further modifications can be made to refine the responsiveness of this system over time, including raising the minimum point thresholds and developing mechanisms to assist state programs that have difficulty meeting those thresholds.

For the 16 states, samples from animals categorized as strange-acting and found dead had a greater chance of being rabid, similar to the analysis of Ohio ERS data for 2005–2007 [9]. The strange-acting sample category resulted in detection of the greatest number of rabid animals; however, due to considerable variability in the ratio among rabid animals and samples tested by category across states, there was a lower predictive value by simple linear regression than expected. Rabid animal predictability for this category may be confounded because strange-acting behaviors may be the result of other neurological diseases. In the future, we will promote an improved and standardized interpretation among WS staff and cooperators as to what constitutes a strange-acting animal. While there was a significant relationship between the category found dead and rabid meso-carnivores detected, additional data over time will be required to determine the ability to predict rabid meso-carnivores beyond the r^2^ = 0.32 generated for 2016 with Michigan data removed as a potential outlier. 

In Ohio during 2005–2007, all other ERS categories resulted in detection of rabid animals, except for samples provided predominantly through NWCO sources (i.e., healthy animals). While our results were consistent with Ohio findings, NWCO-derived positive cases were detected at a slightly higher rate than expected during 2016. Thus, the value of NWCO-collected or otherwise apparently healthy animals should not be totally discounted as a means to find rabid animals. Use of NWCO-provided samples could be important in helping to define the extent of disease spread in a newly emerging rabies focus, in high-risk spread corridors, such as urban-suburban environments that support larger reservoir populations, or in areas where rabies is enzootic, even though only six of 3926 (0.2%) animals within this category were rabid.

Although rabies-positive samples from road-killed animals represented only <1% of all specimens collected in this category, this source of samples remains important because opportunistic and formal road kill surveys may help to fill spatial and temporal ERS gaps. Moreover, the strong relationship between road-killed samples collected and rabid meso-carnivores detected in 2016 suggests future predictive value from this sample category. Road kills typically occur in proportion to population density of the target species and often increase or decrease seasonally relative to activity patterns [15,16,17]. In our seasonal analysis, road-killed animals were the most important source of rabies positive samples during winter, a time of year when rabies case detection is generally lower [18]. Future refinements to the ERS initiative will include improved definitions for opportunistic versus formal road kill surveys, and will better delineate a tiered structure for evaluating road types (e.g. county road, state highway, interstate) that should be surveyed at higher rates based on traffic volume and in areas where samples from other ERS categories are difficult to obtain.

No rabid animals were detected from surveillance-trapped animals during 2016 ERS efforts. However, there were no rabies outbreaks identified where focal trapping efforts were warranted. Targeted surveillance trapping has served as a source of rabid animals in other outbreak situations, but it is highly variable, costly and labor intensive [14,19,20]. In the U.S., the rabies detection rate in targeted surveillance-trapped animals has ranged from 0% in New York and Ohio to an unusually high detection rate of 2.2% in Alabama. Only 0.1% of targeted surveillance-trapped animals in Ohio were detected during 2005, with no rabid animals captured during 2006–2007 [9]. Trapping in response to an index or early identified cases may serve as a useful ERS tool to better define an outbreak focus.However, this form of ERS may be most effective for a transient period of a few weeks in close proximity to such cases in new outbreak locations, as observed in Quebec, Canada [14]. 

Seasonally, rabies was detected most frequently detected during the spring, followed by summer, which is consistent with reported seasonal peaks in other regions [14,18]. Peaks of rabies activity during spring may result from increased denning and mating contacts in winter [21]. Secondary summer peaks may be related to increased contact during juvenile dispersal [14,18,22]. Although total sample collection was similar in spring and summer, samples declined through the fall and winter months. This observation may be related in part to lapses in ERS due to other rabies management priorities in some states during the late summer-fall months (e.g., ORV baiting and post-ORV monitoring efforts). In some northern latitudes, lower sample size during winter months may also be influenced by reduced animal activity during extreme cold and high snowfall. 

To target ERS seasonal activities better, it may be more appropriate to consider biological seasons in future analysis, though pregnancy/parturition, young-rearing, dispersal, and breeding roughly coincide with spring, summer, autumn, and winter, respectively [23]. However, surveillance should not completely cease even at northern latitudes. Strange-acting animals during cold, snowy periods may have a high chance of being rabid, as observed in Franklin County, NY during the winter of 2015, as well as a recent rabies incursion near Hamilton, Ontario, Canada [24]. Latitudinal variation should be considered, as seasonal behavior patterns in raccoons found in more northern latitudes may result in differences in both sample collection and disease detection rates, compared to more moderate winter conditions that occur in the southeastern U.S. [25]. 

This initial study suggests that qualitative stratification of sampling categories provides a more meaningful way to evaluate ERS. However, we expect that data for the first three consecutive years should provide an improved analytical milestone to determine if this ERS system warrants modifications.The value of a revitalized ERS network has been realized in Ohio, where two strange-acting raccoons with no known exposure history submitted through a cooperator network freezer tested positive for the raccoon rabies virus variant during March–April 2017. This prompted a contingency action response because the cases were >8 km (5 miles) beyond the established ORV zone. An emergency ORV spring baiting occurred to eliminate this focus and prevent raccoon rabies from gaining a stronger foothold in east-central Ohio and beyond. Without an established ERS network, both cases would have gone undetected through traditional public health surveillance until a local epizootic emerged that resulted in human or pet exposures. 

Applying point values to public health surveillance data may represent a logical progression to help refine sample category weights, because as expected, exposure-based suspect animals have a greater chance of being rabid based on the Ohio data for 2005–2007. Also, a better evaluation of ERS efforts through space and time may require more refined sample targets using defined spatial resolution and habitat type [14]. Among the pressing surveillance challenges is the need to identify significant risk corridorsin relation to ORV and geophysical ‘barriers’ through which rabies has a higher probability of spreading to naïve areas. Existing models have illuminated potential raccoon rabies spread scenarios [26,27] as well as the value of targeted surveillance to formulate control plans, but developing models that possess predictive sensitivity to improve appropriate rabies management for a common, often ubiquitous, ecological generalist such as the raccoon remains a daunting task. Therefore, continuing to develop effective stratified sampling regimes to guard against spatial-temporal surveillance gaps in relation to a suite of variables, including epidemiologic facets, ecological differences, local host abundance, species behavior, rabies virus variant distribution, viral spillover potential, spatial distribution of rabies collaborators on the landscape, and other factors, will remain critical for successful implementation of wildlife management strategies to eliminate rabies. 

## Figures and Tables

**Table 1:** Examples of expanded enhanced rabies surveillance cooperative network contacts.

Cooperator Level	Cooperator	Recommended Minimum Contacts
Federal	United States Department of Agriculture (USDA) Forest Service, USDA Veterinary Services, U.S. Fish and Wildlife Service, U.S. Department of Energy, U.S. Department of Defense, Tribal Nations	2
State	Health, Agriculture, Transportation, Wildlife/Natural Resources, Parks/Recreation, Police	3
County	Animal Control, Health, Police, Municipal Waste, Transportation, Agriculture Extension, Parks/Recreation	4
City/Town/Hamlet	Local Police, Fire Department, Community Clubs, Homeowners Associations,	3
Special Interest	Nuisance wildlife control operators (NWCO), Hunting/Trapping, Wildlife Rehabilitator, Veterinarians, Hiking/Backpacking Clubs	3
Other	4-H, U.S. Animal Health Association, Zoos, Wildlife and Public Health Professional Societies	No minimum but highly encouraged

**Table 2:** Six standardized sample categories and associated stratified point values (i.e., weights) for enhanced rabies surveillance.

Category	Point Value	Description
Strange-acting	15	Suspect behavior suggestive of neurological disease
Found dead	5	Unexplained with no obvious signs of trauma; not road kill
Road kill	3	Formal survey or opportunistically; 1 additional point/mile driven
Surveillance-trapped	2	Active trapping in specified raccoon rabies risk areas/response to an outbreak
NWCO/other	1	Nuisance-trapped or homeowner-derived; apparently healthy
Unknown	1	Behavior not observed; fate not determined

**Table 3:** Total enhanced rabies surveillance samples collected and tested by Wildlife Services, and total category points assigned, 1 January–31 December 2016.

Sample Category	Point Value	Total Samples (%)	Total Points (%)
1 = Strange-acting	15	1239 (18.1)	18,585 (66.7)
2 = Found dead	5	225 (3.3)	1125 (4.0)
3 = Road kill	3	1370 (20.0)	4110 (14.8)
4 = Surveillance-trapped	2	13 (0.2)	26 (0.1)
5 = NWCO/other	1	3924 (57.3)	3924 (14.1)
6 = Unknown	1	81 (1.2)	81 (0.3)
Totals		6852	27,851

## Supplementary Materials

The following is available online at www.mdpi.com/2414-6366/2/3/34/s1: Figure S1: Call algorithm template for enhanced rabies surveillance.
