# Supplementary material for: Enhanced Rabies Surveillance to Support Effective Oral Rabies Vaccination of Raccoons in the Eastern United States
Source: Trop Med Infect Dis. 2017 Jul 28;2(3):34. doi: 10.3390/tropicalmed2030034 (PMC6082093; doi:10.3390/tropicalmed2030034)
Supplement: Supplementary file 1 [file tropicalmed-02-00034-s001.pdf]

Supplementary Material: Enhanced Rabies Surveillance to Support Effective Oral Rabies Vaccination of Raccoons in the Eastern United States

Jordona D. Kirby, Richard B. Chipman, Kathleen M. Nelson, Charles E. Rupprecht, Jesse D. Blanton, Timothy P. Algeo and Dennis Slate

DID AN EXPOSURE TO RABIES OCCUR?**

BITE: Did animal's teeth penetrate the skin of the patient/pet?

Non-bite: Did animal's saliva or neural tissue make contact with the patient/pet's mucous membranes or broken skin?

Unrecognized (bats only): Did the bat make direct contact with the patient/pet, or was the bat found in a bedroom when the patient was sleeping, or was the bat found in a room with a child or other person unable to communicate potential exposure?

YES →

Contact the state or county Health Department for guidance, or submit animal to the Health Department for testing.

State Department of Health: xxx-xxx-xxxx

County Department of Health: xxx-xxx-xxxx

NO ↓

Is the animal within the WS enhanced surveillance coverage area?
Please see the attached map.

NO →

Dead: Dispose of carcass. (Bury underground 18" to 24".) Do not bury the carcass in a plastic bag.

Alive: Contact local animal control for potential assistance with wild animals and for domestic animals.

The State Department of Health may test the animal for a fee (charged to the landowner).

YES ↓

Is the animal's head (brain) viable for testing?

The carcass should be "fresh" with minimal decay, relatively whole head, and short duration of time animal has been deceased (weather dependent).

Raccoons, skunks, foxes, coyotes and bobcats are high priority!

NO →

Dead: Dispose of carcass. (Bury underground 18" to 24") Do not bury the carcass in a plastic bag.

Avoid direct contact with carcass. (Use shovel or heavy duty rubber gloves!)

YES ↓

Alive: Animal will need to be euthanized
DO NOT SHOOT ANIMAL IN THE HEAD!
Dead: Arrange for collection and transport by calling the nearest WS office for testing. Record contact information and animal collection information (Date, Location, and Address if available)

Sample Preservation →

The sample should be collected as soon as possible! If the sample cannot be frozen, then it needs to be collected within 1–3 days.
Please keep the sample cool by placing it in the shade or storing it in a cooler.
Contact Wildlife Services for guidance:
xxx-xxx-xxxx or 1-866-487-3297

Figure S1. Call Algorithm Template for Enhanced Rabies Surveillance.
